# Safe Placement of Ommaya Reservoirs in Thrombocytopenic Patients: One Institutions Experience

**DOI:** 10.7759/cureus.5291

**Published:** 2019-07-31

**Authors:** Kimberly Major, Abraham Schlauderaff, Amalia Brawley, David E Hale, Elias Rizk

**Affiliations:** 1 Neurosurgery, Penn State Milton S. Hershey Medical Center, Hershey, USA; 2 Neurosurgery, Penn State College of Medicine, Penn State Milton S. Hershey Medical Center, Hershey, USA

**Keywords:** ommaya, thrombocytopenia, stereotactic, hemorrhage

## Abstract

Objective

The purpose of this study was to assess the risk of hemorrhagic complications in thrombocytopenic patients after Ommaya reservoir placement.

Methods

Between 2009 and 2017, 192 patients were identified on the National Neoplastic Meningitis Registry and had undergone Ommaya reservoir placement for intrathecal chemotherapy. A retrospective chart review was performed to collect the preoperative and postoperative platelet levels, whether or not the patient received any transfusion of platelets, neurological exams, and whether a postoperative head CT was obtained. Using generally accepted recommendations, a platelet level less than 100,000/μL was considered clinically significant and used as our threshold for thrombocytopenia.

Results

Seven patients (3.6%) were identified as thrombocytopenic in our patient population with platelet counts ranging from 54,000 to 99,000/μL. Primary diagnoses for the seven patients included leukemia, prostate cancer, primary brain cancer (four patients), and lung cancer (non-small-cell lung carcinoma). One patient received platelet transfusions preoperatively. Three patients had a routine head CT obtained postoperatively with no abnormal findings noted. There were no changes in the neurological exam noted in all of the patients included in this study. No clinically significant hemorrhages were identified in our patients.

Conclusions

From our single institutional experience, we found that thrombocytopenia is fairly uncommon, found in only 3.6% of our patients undergoing placement of Ommaya reservoirs. We did not encounter any increased risks of postoperative hemorrhage in studied thrombocytopenic patients.

## Introduction

Dr. Ayub Ommaya, a pioneering neurosurgeon, originally started placing subcutaneous cerebrospinal fluid reservoirs in 1962 and soon thereafter published his results [1]. This procedure consists of passing a ventricular catheter into the lateral ventricle through a burr hole. The catheter is then attached to a subcutaneous reservoir, which may be accessed via subsequent needle punctures. Since its conception, the Ommaya reservoir has developed widespread usage for treatment of various forms of intracranial malignancies. There are potential, well-described complications associated with the Ommaya reservoir, including aseptic meningitis, catheter-related infection, catheter malplacement, wound dehiscence, and leakage of cerebrospinal fluid [2, 3]. However, there is scant literature describing intraparenchymal hemorrhage risk associated with placement of the Ommaya reservoir. A National Surgical Quality Improvement Program analysis by Dasenbrock et al. found moderate (100,000-124,000/µL) and severe (75,000-99,000/µL) thrombocytopenia were associated with mortality and reoperation after craniotomy for tumor [4]. Field et al. described their experience of intracerebral hemorrhage after stereotactic biopsy in five hundred patients, which showed the only statistically significant factor was the degree to which the platelet count fell below 150,000/µL [5]. Kennedy et al. reported a hemorrhagic complication rate of 6.4% (seven out of 109 patients) after stereotactic catheter placement for Ommaya reservoirs, with one patient experiencing preoperative thrombocytopenia [6].

Ommaya reservoirs are commonly placed for chronic administration of intrathecal chemotherapy in various disease states such as neoplastic meningitis or central nervous system (CNS) lymphoma. Not uncommonly, patients may have concurrent systemic disease processes including disorders of myelopoiesis, prohibiting safe placement of Ommaya reservoirs. Thrombocytopenia is often considered a contraindication to neurosurgical procedures, and the current expert opinion is that a platelet count threshold of 100,000/μL should be used to decrease the risk of hemorrhagic complications [7]. However, the concept of a "safe" platelet count lacks evidence-based recommendations and demonstrates great variability between patients even with the same disorder. Current hematologic guidelines suggest an increased risk of surgical bleeding with platelet counts less than 50,000/μL in major surgery and invasive procedures such as lumbar puncture, liver biopsy, endoscopy with biopsy, and placement of central venous catheter [8]. For high-risk procedures operations to critical sites, eye surgery, and neurosurgery, it is suggested that a platelet count of less than 100,000/μL be obtained before proceeding with surgery [8]. In the present study, we describe our single institution’s experience of safely placing Ommaya reservoirs in thrombocytopenic patients.

## Materials and methods

Since 2011, all Penn State Health patients that had Ommaya reservoirs placed for neoplastic meningitis were included in the National Neoplastic Meningitis Registry. A retrospective review of all patients in the registry was performed to collect the following data: preoperative and postoperative platelet levels, whether or not the patient received any transfusion of platelets, change in the neurological exam, and whether a postoperative head CT was obtained. The discrepancy between pre- and postoperative neurological exams was queried to ascertain a clinically relevant hemorrhage. Cranial imaging, e.g. head CT, when available, was also reviewed to assess for intra-parenchymal hemorrhage presence and severity. Using generally accepted recommendations, we determined that a platelet level less than 100,000/μL was clinically significant and thus was used as our threshold for thrombocytopenia. Patient data was de-identified and stored using Research Electronic Data Capture (REDCap).

## Results

There were a total of 192 patients entered in the National Neoplastic Meningitis Registry at the time this study took place. Surgeries were performed between 2009 and 2017. Seven patients (3.6%) were identified to have a platelet count less than 100,000/μL (ranging 54,000-99,000/μL). Primary diagnoses for the seven patients included leukemia, prostate cancer, primary brain cancer (four patients), and lung cancer (non-small-cell lung carcinoma). One patient received platelet transfusions preoperatively to correct a level of 45,000/μL which was deemed too low by the primary surgeon. Three patients had a routine head CT obtained postoperatively with no abnormal findings noted. There were no changes in the neurological exam noted in all of the patients included in this study (Table 1).

**Table 1:** Breakdown of surgical patients' platelet counts, transfusion, neurological and CT status

Patient	Preop Platelet Level (K/uL)	Postop Platelet Level (K/uL)	Transfusion Given	Neurological Change	CT Performed
1	99	97	No	No	No
2	89	97	Yes	No	Yes - Normal
3	84	68	No	No	Yes - Normal
4	94	85	No	No	Yes - Normal
5	85	114	No	No	No
6	77	102	No	No	No
7	54	121	No	No	No

## Discussion

There is limited data in the literature regarding safe passage through the brain parenchyma in patients with thrombocytopenia. Dasenbrock et al. previously found that the strongest predictor of any cranial reoperation after craniotomy for tumor was preoperative thrombocytopenia [4]. The results of our study indicate that there is no increased risk of postoperative hemorrhage with preoperative thrombocytopenia during placement of Ommaya catheters, contrary to Field et al. findings in stereotactic biopsies [5]. These findings may be applicable to other forms of targeted stereotactic procedures such as ventricular shunt placement, and further research is needed in this area. This study is limited by its relatively small sample size, the lack of a control group and its retrospective nature. Furthermore, variability in stereotactic equipment used among different neurosurgery teams, patient demographics and comorbidities, and surgical technical skills make it difficult to generalize our results to all candidates for Ommaya reservoirs.

## Conclusions

From our single institutional experience, we found that thrombocytopenia is uncommon, found in 3.6% of our patients undergoing placement of Ommaya reservoirs. We did not encounter any clinically relevant hemorrhage in our thrombocytopenic patients. We conclude that the benefits of Ommaya reservoir placement outweigh the risks associated with thrombocytopenia in the neoplastic meningitis population. As always, close monitoring of platelet counts pre and postoperatively is imperative to the safety and efficacy of neurosurgical procedures in thrombocytopenic patients.
